# A kinase inhibitor screen identifies signaling pathways regulating mucosal growth during otitis media

**DOI:** 10.1371/journal.pone.0235634

**Published:** 2020-08-06

**Authors:** Julia Noel, Keigo Suzukawa, Eduardo Chavez, Kwang Pak, Stephen I. Wasserman, Arwa Kurabi, Allen F. Ryan

**Affiliations:** 1 Department of Surgery/Otolaryngology, UC San Diego, San Diego, CA, United States of America; 2 School of Medicine, UC San Diego, San Diego, CA, United States of America; 3 San Diego VA Healthcare System, San Diego, CA, United States of America; Wayne State University, UNITED STATES

## Abstract

Otitis media, the most common disease of childhood, is characterized by extensive changes in the morphology of the middle ear cavity. This includes hyperplasia of the mucosa that lines the tympanic cavity, from a simple monolayer of squamous epithelium into a greatly thickened, respiratory-type mucosa. The processes that control this response, which is critical to otitis media pathogenesis and recovery, are incompletely understood. Given the central role of protein phosphorylation in most intracellular processes, including cell proliferation and differentiation, we screened a library of kinase inhibitors targeting members of all the major families in the kinome for their ability to influence the growth of middle ear mucosal explants *in vitro*. Of the 160 inhibitors, 30 were found to inhibit mucosal growth, while two inhibitors enhanced tissue proliferation. The results suggest that the regulation of infection-mediated tissue growth in the ME mucosa involves multiple cellular processes that span the kinome. While some of the pathways and processes identified have been previously implicated in mucosa hyperplasia others are novel. The results were used to generate a global model of growth regulation by kinase pathways. The potential for therapeutic applications of the results are discussed.

## Introduction

Otitis media (OM) is the most common childhood disease, affecting more than 90% of children under five years of age [1]. OM causes more physician visits, prescriptions, and surgeries than any other pediatric condition. The estimated annual cost of OM in the US is $6 billion in health care and associated expenses [2,3]. The peak of OM incidence also occurs at a critical developmental period. Chronic OM has been associated with delays in speech and language and learning [4–6]. In many developing countries, OM is a very serious health issue. The World Health Organization (2004) estimates that undertreated OM causes ~30,000 annual deaths and 50% of the world’s serious hearing loss [7].

Antibiotics are no longer recommended for uncomplicated acute OM (AOM), or OM with effusion, in children over two [8]. Despite this recommendation, treatment patterns have shown little change due largely to parental demand for intervention [9]. Antibiotic therapy is still recommended for children under two [10], and for complicated forms of OM with more severe symptoms [11]. While Streptococcal vaccines have reduced OM from the covered strains, increased incidence of other strains and microbes have largely offset these gains [12,13]. Ventilation tubes can be effective for recurrent or chronic OM, but there is controversy about long-term benefit and later tympanic membrane (TM) abnormalities such as scarring or tympanosclerosis [14,15]. Given the above issues, alternative treatments for OM are needed.

A major pathologic feature of OM is hyperplasia of the mucosa lining the middle ear (ME), in which the normal monolayer of simple squamous epithelial cells converts to a thickened and respiratory-type epithelium. This includes a greatly expanded, semi-stratified, cuboidal epithelium with goblet and ciliated cells as well as a robust stroma. Hyperplasia contributes to the deleterious sequelae of OM, including mucous secretion into the ME lumen [16]. Mucosal neovascularization also increases the opportunity for leukocytic infiltration, tissue edema and ME effusion. Expansion of the stroma, with their activation of stromal cells, may lead to fibrosis and bone erosion that can permanently damage the sound conduction apparatus of the ME, resulting in persistent hearing loss. A study of Danish teenagers reported that 15–20% exhibited visible scarring of the tympanic membrane due to prior OM [17]. While mucosal growth is an important aspect of OM, the intracellular processes that mediate ME hyperplasia are not well understood. A better comprehension of OM pathophysiology may lead us toward potential new therapies for this disease.

It is well known that intracellular signaling plays a critical role in tissue growth. We and others have identified several pathways involved in ME mucosal hyperplasia (e.g. [18–20]). using cell signaling inhibitors important in other systems. However, no signaling pathway operates in isolation. Rather, cell signaling consists of a network of pathways and processes, operating to integrate multiple inputs in determining cellular outcomes. Given this degree of complexity, it is likely difficult, if not impossible, to identify all of the various cellular processes that contribute to ME mucosal hyperplasia by *a priori* reasoning. A solution to this complexity employed in other systems is high-throughput screening. A broad screen could potentially identify novel pathways involved in hyperplasia that might not otherwise be obvious candidates. High throughput screening is typically performed *in vitro*. Immortalized cell lines have been created from the ME mucosa [21], that could be used for such a screen. However, cell lines are typically quite different from primary tissue. As a compromise, we used explants of the rat ME mucosa to create a medium-throughput screening method.

**Fig 1:**
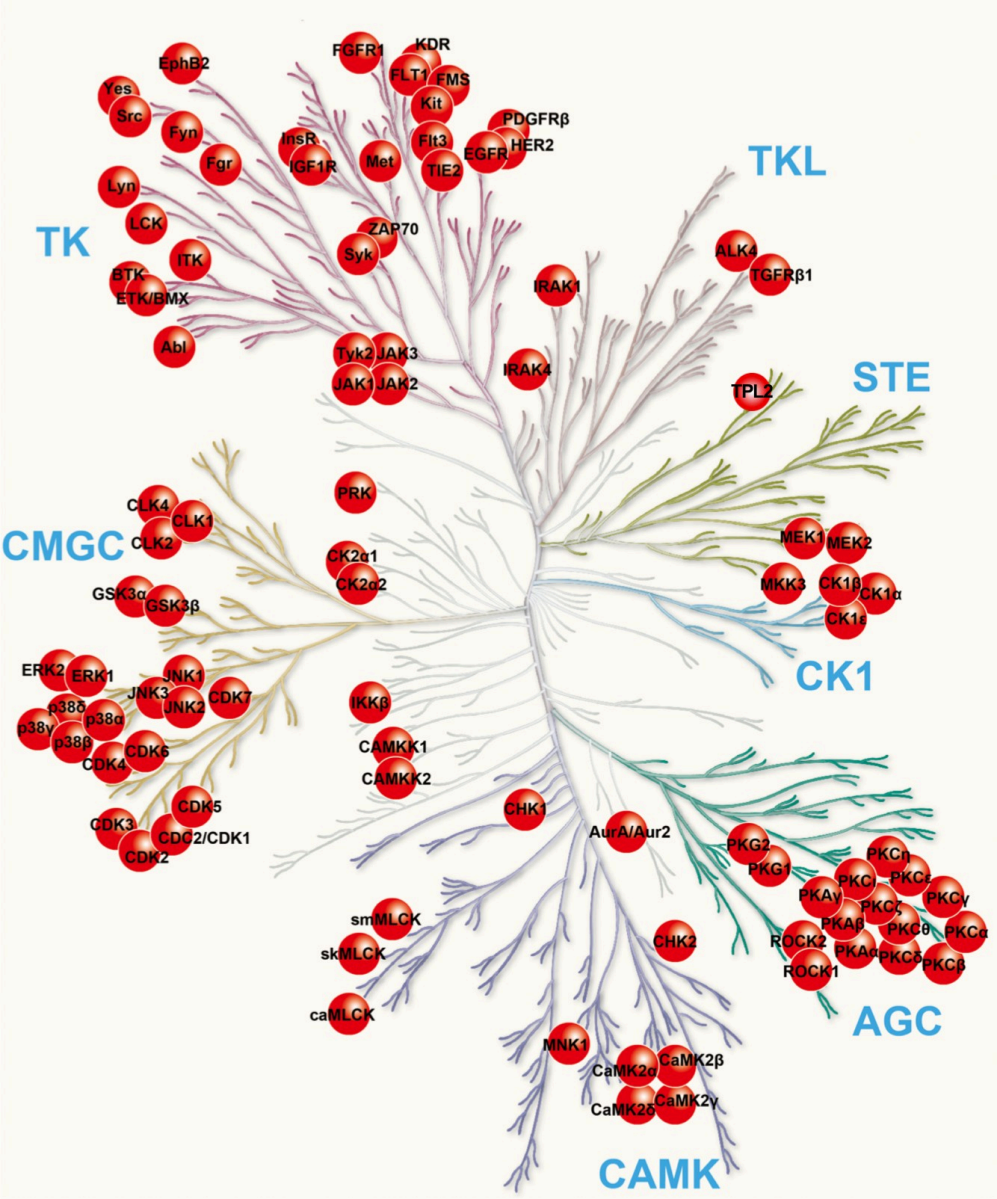
A phylogenetic tree depicting the kinome, members of the superfamily of mammalian protein kinases. The kinases targeted by inhibitors in the libraries employed in this study are indicated by red circles. *Kinase families*: AGC: containing the PKA, PKG, PKC families; CAMK: Calcium/calmodulin-dependent protein kinases; CK1: Cell kinase 1 group; CMGC: containing the CDK, MAPK, GSK3 and CLK families; STE: Serine-threonine kinases, homologs of yeast sterile kinases; TK: Tyrosine kinases; TKL: Tyrosine kinase–like family. The kinome diagram is adapted, with permission, from Cell Signaling Technologies.

Since ME mucosal hyperplasia is highly stereotypical, it is presumably tightly regulated. In uncomplicated, acute OM the mucosa usually recovers completely after resolution, which would involve additional regulatory pathways. Many signaling pathways involve protein phosphorylation by kinases, which alters the functional state of the protein target. Kinase inhibitors are thus a major research tool used to identify regulatory pathways. They have been employed to study several mucosal diseases, primarily those related to neoplastic or dysregulated growth (e.g. [22]). For this study, we evaluated a library of 160 kinase inhibitors, with targets distributed throughout all known kinases (collectibelym referred to as the kinome) (Fig 1), for their ability to regulate the growth of bacterially infected ME mucosa.

## Materials and methods

### Animals

For *in vitro* studies, subjects were 60–90 day old Sprague Dawley rats (Harlan). For gene array studies, subjects were C57/WB F1 mice (Jackson Labs). Animals were housed under specific pathogen-free conditions. All experiments were performed to National Institutes of Health guidelines and approved by the Institutional Animal Care and Use Committee of the VA San Diego Medical Center.

### Bacteria

*Haemophilus influenzae* strain 3655 (nontypeable, biotype II), which had been = isolated from the ME of an OM patient in St. Louis, was provided by Dr. Asa Melhus, Lund University, Sweden, was used at titers of 5x102-10^3^ to induce infection of the ME. Inocula were prepared as described earlier [23,24].

### Surgery

All surgeries were performed under standard aseptic conditions. Animals were deeply anesthetized with rodent cocktail (10 mg/kg xylazine, 1 mg/kg acepromazine and 50 mg/kg ketamine i.m.). For *in vitro* studies, and approximately 50 μl of NTHi inoculum was bilaterally injected into the ME of rats via the surgically exposed ME bulla, after which the incision was closed. Following inoculation, the tympanic membranes were visually confirmed to be intact. After 48 hours, rats were anesthetized and humanely sacrificed. The ME bullae were harvested and opened. The ME mucosa was carefully dissected from the hypo-tympanum as a single sheet. The tissue was placed in a sterile petri dish with a layer of black silastic rubber on the bottom, in PBS. A diamond scalpel was used to cut the mucosa into approximately 0.25 x 0.25 mm explants.

### Kinase inhibitor libraries

The kinase inhibitory compounds used (EMD Calbiochem Kinase Inhibitor Libraries) were contained in two separate libraries (I and II), each consisting of 80 inhibitory compounds. The libraries broadly targeted elements of the mammalian kinome, with inhibitors against all of the major kinase families, as shown in Fig 1. The libraries were provided in two separate sets of 96-well plates (I and II), and all compounds were provided in DMSO. Each library set included both DMSO and blank wells for controls.

### Tissue culture

The explants were placed individually, lumen-side up, into 48-well culture plates (Co-Star). A 150 μl of culture medium (75% DMEM, 25% Hams-F12 supplemented with 5% bovine serum, with additives: 100 IU/mL penicillin, 100 mg/mL streptomycin, 0.4 mg/mL hydrocortisone and 10^−6^ M isoproterenol) was carefully added so that the fluid meniscus kept the explant flat on the well bottom. The explants were cultured for 24 hours to allow for attachment. The culture medium was then increased to 250 μl and the explants cultured for two more days to facilitate adhesion and expansion.

After, the mucosal explants were randomly divided into groups of three explants per concentration, and three concentrations of each kinase inhibitor were applied. The 160 kinase inhibitors from libraries I and II were diluted in warm culture media and added to explant wells at concentrations of 0.05, 0.5, or 5.0 μM. For each plate, one group of 3–6 explants/plate was cultured without an inhibitor but with DMSO (0.1% concentration, as used for all inhibitors after dilution) to serve as negative controls. Kinase inhibitors were applied from 3–9 days *in vitro* (DIV) for a total of six days. Each explant was then fixed and photographed, and the 9 DIV area of mucosa growth and proliferation was measured using NIH SPOT Image software.

### Data analysis

Preliminary studies were performed using different numbers of control explants and explants treated with 0.5 μM of the JNK inhibitor SP000126, which we had previously found to reduce explant growth [20]. These prior studies established that approximately 8–10 explants would be required to identify a statistically significant difference due to a given treatment. As this number was not practical for a screen, we used an N of three and applied threshold measures to select compound hits. For mucosal samples treated with 0.5 μM of an inhibitor, a criterion threshold of <10 mm^2^ after 9 DIV was used. For 5.0 μM, a threshold of <5 mm^2^ was applied. A criterion threshold of >45 mm^2^ was used to identify compounds that increased mucosal growth in culture.

### Gene arrays

To assess the expression of Hippo pathway genes during OM, we employed gene arrays in a mouse model of acute ME infection, as described previously [25]. Briefly, the MEs of C57/WB F1 mice were inoculated with nontypeable Haemophilus influenzae (NTHi) and ME tissue harvested from deeply anesthetized (rodent cocktail) animals at 0 hours (0h, without infection), 3 hours (3 h), 6 h, 1 day (1d), 2d, 3d, 5d or 7d. mRNA from 20 mice at each time point was reverse transcribed and biotinylated cRNA probes were hybridized to duplicate Affymetrix MU430 2.0 microarray sets. The arrays contain ~55,000 probes against virtually all murine genes and including many splice variants. An additional 20 mice at each time point was used to generate a biological replicate. Gene expression levels were evaluated for differences due to infection using variance-modeled posterior inference (VAMPIRE), which distinguishes signal from noise by modeling error structure and identifying expression-related and expression-unrelated variance [26]. Based on the results obtained with inhibitors, expression of genes related to the Hippo pathway in uninfected MEs was compared to that in NTHi-infected MEs at each time point.

### Ethical approval

All applicable international, national, and/or institutional guidelines for the care and use of animals were followed. All experiments were performed to National Institutes of Health guidelines and approved by the Institutional Animal Care and Use Committee of the VA San Diego Medical Center.

## Results

### Controls

When combined across all library plates, negative control explants not treated with any inhibitors exhibited an average area of 25.42 ±0.92 mm^2^ after 9 DIV. Positive control explants, treated with 0.5 μM of the JNK inhibitor SP000126 from 3 DIV to 9 DIV averaged 8.86 ±0.92 mm^2^.

### Library I

**Fig 2:**
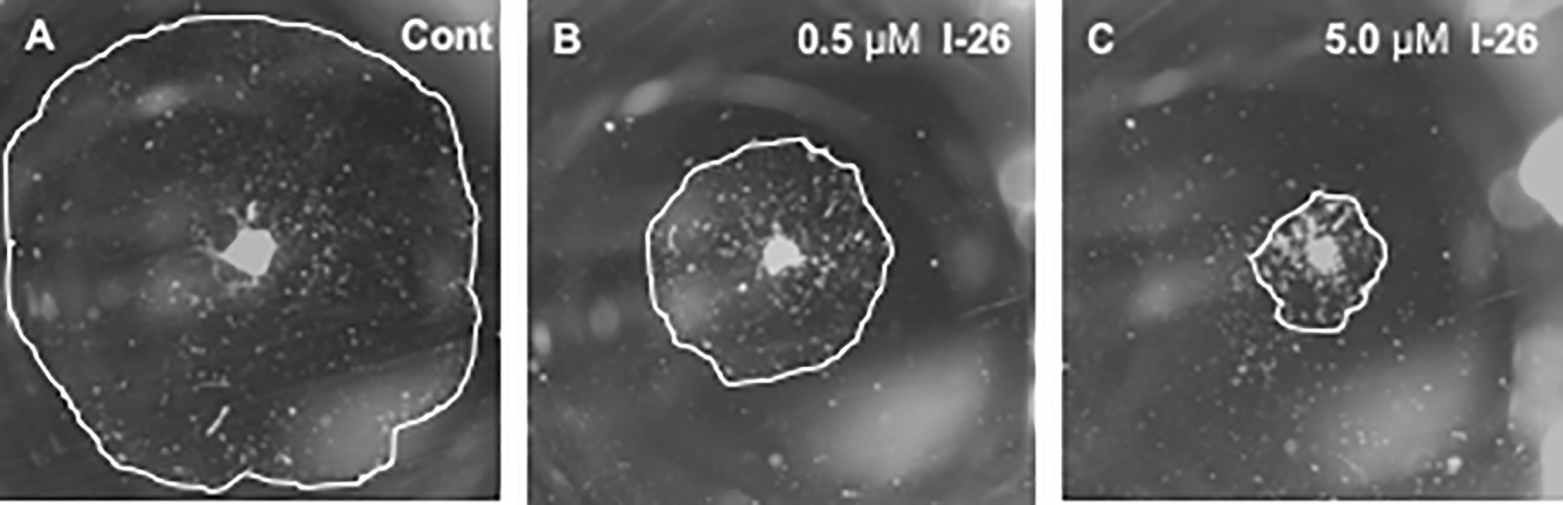
ME mucosal explant cultures. Representative examples of mucosal explants from the MEs of rats. The figure illustrates the growth of explants from rat MEs after 9 days *in vitro* (DIV). A. Negative control explant (not treated with any inhibitor) exposed only to DMSO (Control). B. Explant treated with 0.5 μM Library I compound 26, an EGFR inhibitor, from day 3 to day 9. C. Explant treated with or 5.0 μM Library I compound 26. The white line tracks the edge of each explant, and defines the explant area.

**Fig 3:**
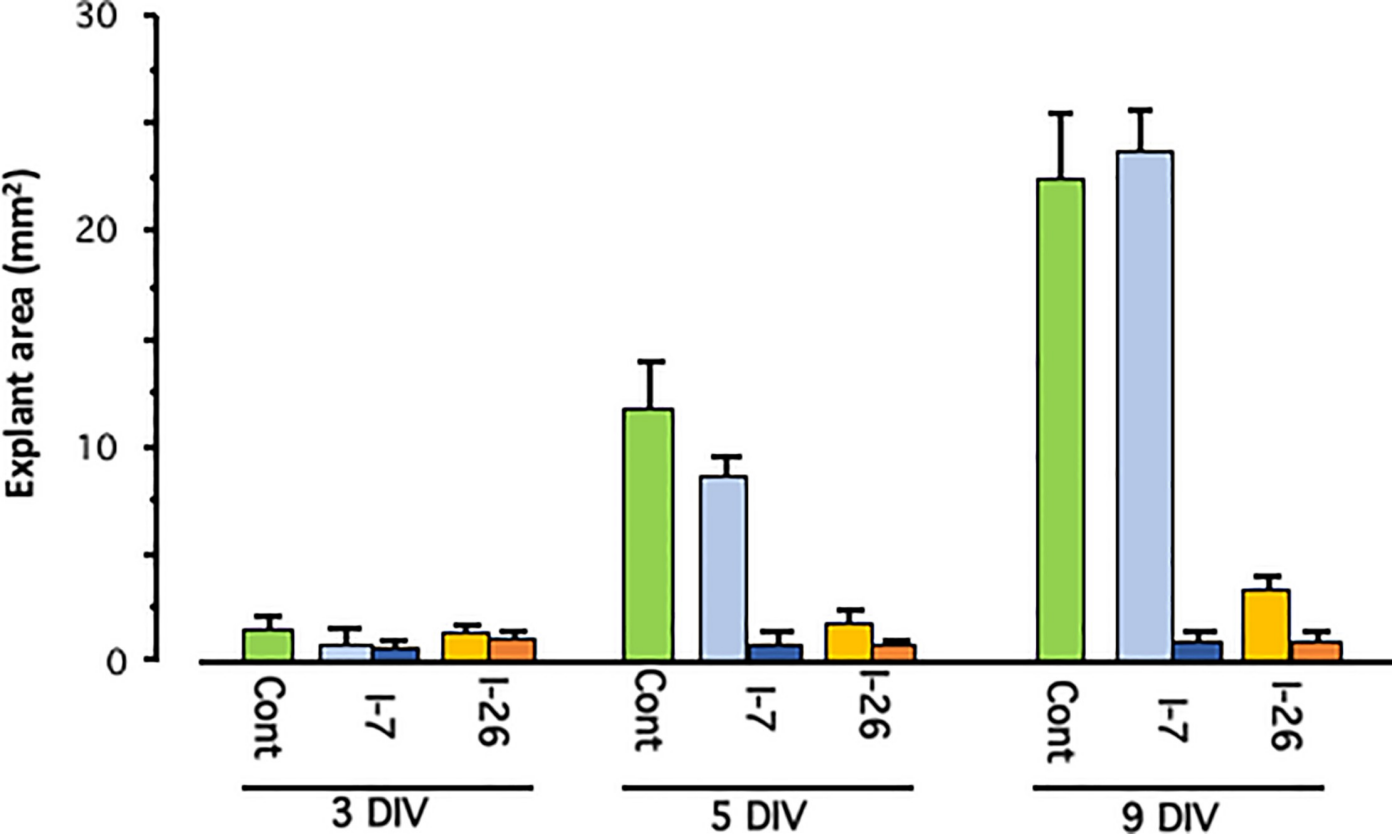
ME mucosal explant growth over time. Average explant areas after 3 DIV, the time at which inhibitors were added, and after 5 or 9 DIV. Negative control explants (green bars) showed steady growth over the culture period. Explants treated with 0.5 μM of Library I compound 7 (light blue bars), an AKT inhibitor, did not show reduced growth. However, explants treated with 5.0 μM (dark blue bars) failed to grow. Explants treated with Library I compound 26 at 0.5 μM (light orange bars) or 5.0 μM (dark orange bars) showed reduced growth compared to controls.

Representative cultures illustrating a control explant and explants treated with two doses of compound 1–26 (an EGFR Inhibitor) from Kinase Inhibitor Library I are presented in Fig 2. Next Fig 3 illustrates average areas at 3 DIV, when inhibitor treatment was started, at 6 DIV and at 9 DIV, for control explants (green bars) as well as explants treated with Library I compound I-7 (AKT Inhibitor X) at 0.5 μM (dark blue) or 5.0 μM (dark blue) or compound I-26 at 0.5 μM (light orange) or 5.0 μM (dark orange). I-7 inhibited growth only at the highest dosage, while I-26 inhibited at both dosages.

**Fig 4:**
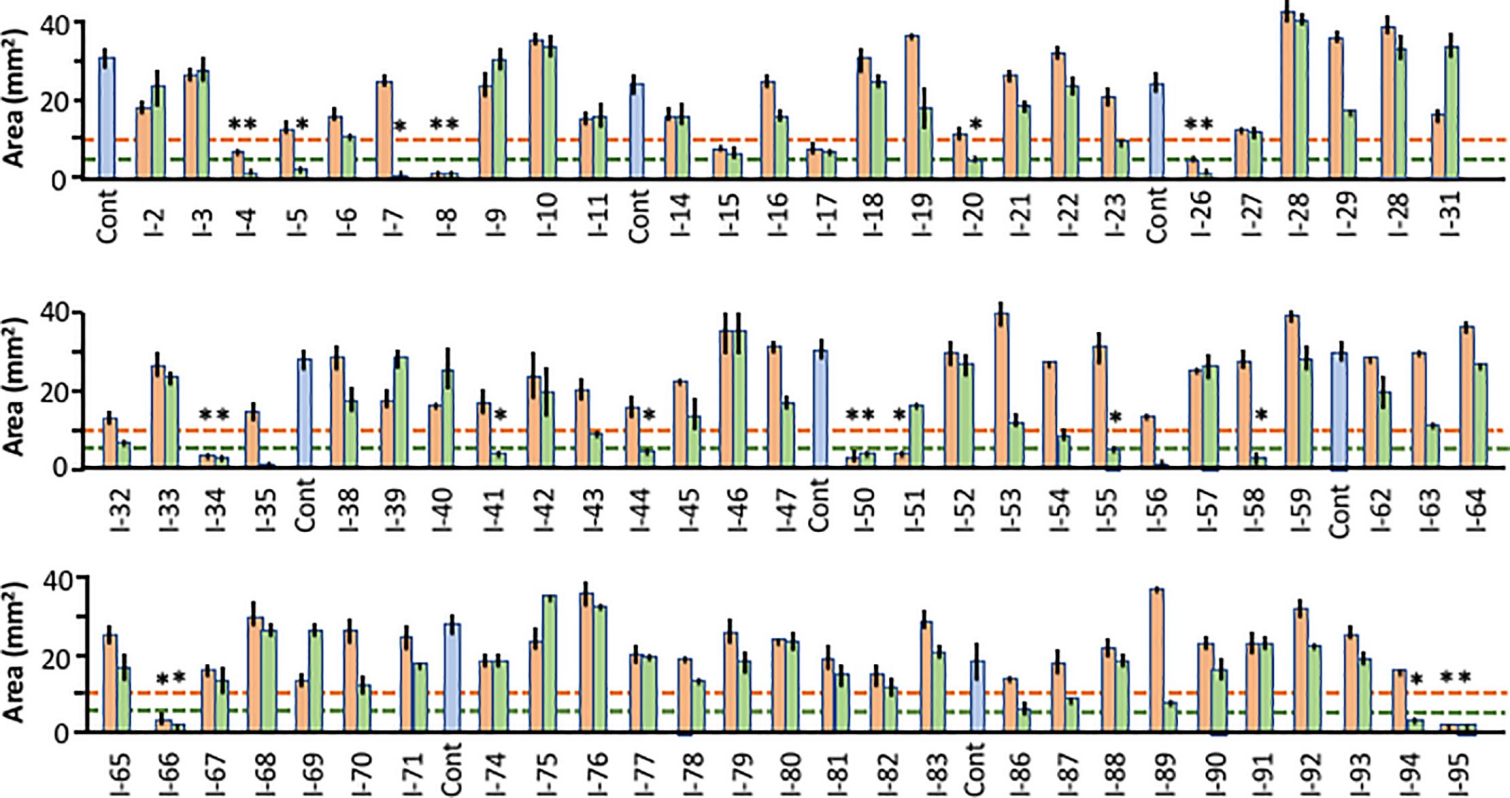
Effects of Library I inhibitors. Explant areas at 9 DIV for Library I, showing areas for control, explants (DMSO alone, blue bars) and inhibitors at concentrations of 0.5 (orange bars) and 5.0 μM (green bars). The criterion threshold of growth suppression for each inhibitor concentration is shown as a dashed line. Inhibitors and concentrations that met the criterion thresholds are indicated by asterisks. All Library I inhibitors and their targets are listed in Table 1.

The results obtained with all compounds present in Library I are illustrated in Fig 4, which shows average explant areas at 9 DIV for two dosages of each inhibitor, plus controls. In this library, 8 compounds reached the criterion threshold for growth inhibition at 0.5 μM of <10mm^2^. These were I-4, AKT Inhibitor IV; I-8, PDK (Pyruvate dehydrogenase kinase)/AKT/Flt(VEGF receptor) Dual Pathway Inhibitor; I-26, EGFR (epithelial growth factor receptor) Inhibitor; I-34, Herbimycin A, inhibitor of Src oncogenes and Iκκb (inhibitor of κb kinase); I-50, PD158780 (EGFR inhibitor); I-51, PD174265 (EGFR inhibitor); I-66, Rapamycin, MTOR (mammalian target of rapamycin) inhibitor and I-95, Staurosporin (broad kinase inhibitor). All of these inhibitors except I-51, PD174265, were also below the criterion threshold at 5.0 μM of <5mm^2^.

Eight additional inhibitors were also below the 5.0 μM criterion threshold, and identified as hits: I-5 and I-7, AKT inhibitors V and X; I-20, PI-103. PI3K (phosphatidylinositol-3 kinase) inhibitor); I-41, JAK3 (Janis kinase 3) Inhibitor II; I-44, LCK (lymphocyte specific protein kinase) Inhibitor; I-55; PDGFR (platelet-derived growth factor receptor) Inhibitor; I-58; PI3K gamma Inhibitor; and I-94, Aurora kinase inhibitor. The identities and targets of all Library I inhibitors are presented in the S1 Table.

### Library II

**Fig 5:**
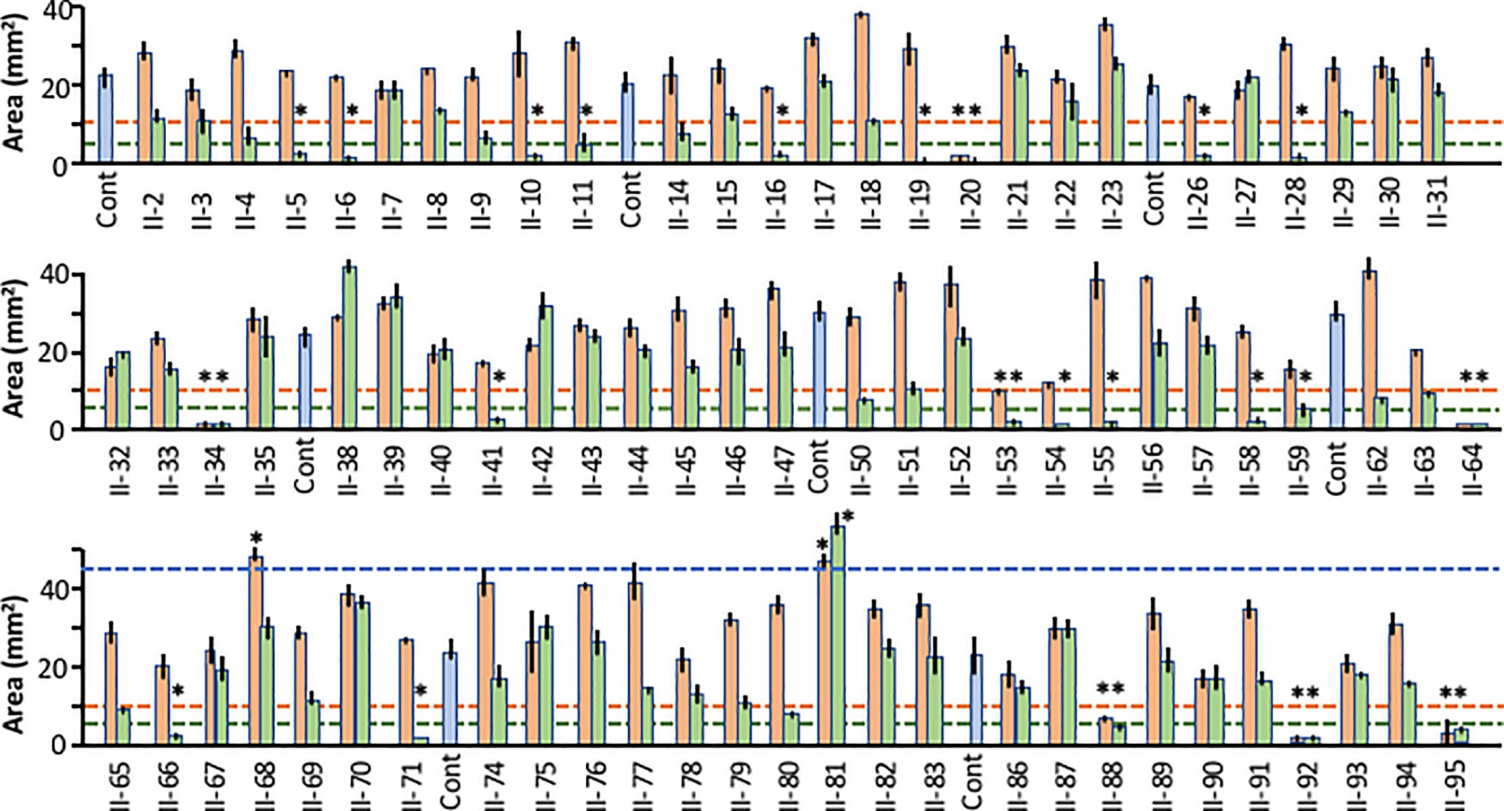
Effects of Library II inhibitors. Explant areas at 9 DIV for Library II, showing areas for control, explants (DMSO alone, blue bars) and inhibitors at concentrations of 0.5 (orange bars) and 5.0 μM (green bars). The criterion threshold of growth suppression for each inhibitor concentration is shown as a dashed line. The criterion threshold for growth enhancement, induced by only two inhibitors, is indicated by a dashed blue line. Inhibitors and concentrations that met the criterion thresholds are indicated by asterisks. All Library II inhibitors and their targets are listed in Table 2.

The results for all Library II compounds are illustrated in Fig 5. In this library, 7 compounds reached the threshold criterion for inhibition at 0.5 μM: II-20, Cdk/Crk Inhibitor; II-34; Cdk1/2 Inhibitor III; II-53, IC261 (casein kinase inhibitor); II-64, K252a (broad kinase inhibitor and staurosporine analog); II-88, SB218078 (checkpoint kinase 1 inhibitor); and II-95, Staurospaurine (broad kinase inhibitor). All were also positive at 5.0 μM.

An additional 15 inhibitors were positive only at 5.0 μM: II-5, Alsterpaullone (broad CDK and Gsk3ß inhibitor); II-6, Alsterpaullone 2-Cyanoethyl (Cdk1 and GSK-3 ß inhibitor); II-10, Compound C (KDR/VEGFR2, ALK2/BMPR-I, and AMPK inhibitor), II-11, Aurora Kinase Inhibitor III; II-16, BAY11-7082, NFκB Inibitor; II-19, CGP74514A (Cdk1 inhibitor); II-26 and II-28, Cdk4 Inhibitors 1 and III; II-41; Fascaplysin (Cdk4 inhibitor); II-54, Iκκ2 Inhibitor IV; II-55, Indirubin Derivative E804 (Stat-3 inhibitor, Cdc2, Cdk2 and Src inhibitor); II-58 and II-59, JNK Inhibitors V and IX; II-66, KN-93 (CaMK-II inhibitor) and II-71, NF-κB activation inhibitor. In addition, explant areas exceeded the threshold for growth enhancement for two Library II inhibitors: II-68, MEK inhibitor II; and II-81, H-89 (PKA inhibitor). The identities and targets of all Library II inhibitors are presented in the Table 1.

**Table 1:** Targets of kinase inhibitor hits in this screen.

Kinome Family	AGC	CAMK	CK1	CMGC	TK	OTHER
					
**Family Members**	AKT	CAMKII	CK1	Cdk1/B	EGFR	PKR
	PKA	MLCK		Cdk2/A	ErbB	DNA-PK
	PKC	CHK1		Cdk4/D1	VEGFR	Aurora-A
	PKG			Cdk6/D1	PDGFR	IKK-2
				JNK1	PDK1	mTOR
				JNK2	Flt3	p70S6K
				JNK3	PI3K	GP140
				GSK-3b	JAK3	TPL2
				p38	IGF-2	
					Syk	
					Lck	
					Kit	
					Fgr	
					Src	

**Table 2:** Kinase inhibitor libraries I and II.

**Library I**	
I-2	AG1024 (IGF1R)
I-3	AGL2043 (IGF1R)
I-4	AKT Inhibitor IV
I-5	AKT Inhibitor V, Triciribine
I-6	AKT inhibitor VIII (Akt1/2)
I-7	AKT Inhibitor X
I-8	PDK/AKT/Flt Dual Pathway Inhibitor
I-9	Aurora Kinase Inhibitor II
I-10	Bcr-Abl Inhibitor
I-11	Bisindolylmaleimide I (GSK3)
I-14	Bisindolylmaleimide IV (PKC)
I-15	BPIQ-I (EGFR)
I-16	Chelerythrine Chloride (PKC)
I-17	Compound 56 (EGFR)
I-18	DNA-PK Inhibitor II
I-19	DNA-PK Inhibitor III
I-20	PI-103 (PI3K)
I-21	Diacylglycerol Kinase Inhibitor II
I-22	Diacylglycerol Kinase Inhibitor II
I-23	EGFR/ErbB-2 Inhibitor
I-26	EGFR Inhibitor
I-27	EGFR/ErbB-2/ErbB-4 Inhibitor
I-28	Flt-3 Inhibitor
I-29	Flt-3 Inhibitor II
I-30	cFMS Receptor TK Inhibitor
I-31	Gö 6976 (PKC)
I-32	Gö 6983 (PKCα,PKCβ)
I-33	GTP-14564 (Class III Receptor TK)
I-34	Herbimycin A (P50 v-src)
I-35	Flt-3 Inhibitor III
I-38	IGF-1R Inhibitor II
I-39	IRAK-1/4 Inhibitor
I-40	JAK Inhibitor I
I-41	JAK3 Inhibitor II
I-42	JAK3 Inhibitor II
I-43	JAK3 Inhibitor II
I-44	Lck Inhibitor
I-45	LY294002 (PI3K)
I-46	LY303511 (neg. control, LY294002)
I-47	Met Kinase Inhibitor
I-50	PD158780 (ErbB)
I-51	PD174265 (EGFR)
I-52	PDGF Receptor TK Inhibitor II
I-53	PDGF Receptor TK Inhibitor III
I-54	PDGF Receptor TK Inhibitor IV
I-55	PDGF RTK Inhibitor
I-56	PKR Inhibitor
I-57	PKR Inhibitor, Negative Control
I-58	PI 3-Kg Inhibitor
I-59	PI 3-Kb Inhibitor II
I-62	PP3 (EGFR)
I-63	PP1 Analog II, 1NM-PP1 (P60 c-src)
I-64	PKCbII/EGFR Inhibitor
I-65	PKCb Inhibitor
I-66	Rapamycin (mTOR)
I-67	Rho Kinase Inhibitor III, Rockout
I-68	Rho Kinase Inhibitor IV
I-69	Staurosporine N-benzoyl- (multiple kinases)
I-70	Src Kinase Inhibitor I
I-71	SU11652 (Receptor TK)
I-74	Syk Inhibitor
I-75	Syk Inhibitor II
I-76	Syk Inhibitor II
I-77	TGF-b RI Kinase Inhibitor
I-78	TGF-b RI Inhibitor III
I-79	AG 9 (inactive control)
I-80	AG 490 (EGFR/JAK2)
I-81	AG 112 (EGFR)
I-82	AG 1295 (PDGFR)
I-83	AG1296 (PDGFR)
I-86	AG 1478 (EGFR)
I-87	VEGF Receptor 2 Kinase Inhibitor I
I-88	VEGF Receptor TK Inhibitor II
I-89	VEGFR TK Inhibitor IV
I-90	VEGFR2 Kinase Inhibitor II
I-91	VEGFR2 Kinase Inhibitor III
I-92	VEGFR2 Kinase Inhibitor IV
I-93	DNA-PK Inhibitor V
I-94	Aurora Kinase Inhibitor III
I-95	Staurosporine, Streptomyces sp.—(multiple kinases)
**Library II**	
II-2	KN-62 (CaM kinase II)
II-3	ATM Kinase Inhibitor
II-4	ATM/ATR Kinase Inhibitor
II-5	Alsterpaullone (CDK1/2/5)
II-6	Alsterpaullone, 2-Cyanoethyl (CDK1/GSK3β)
II-7	Aloisine A, RP107 (CDK1/2)
II-8	Aloisine, RP106 (CDK1/GSK3)
II-9	Aminopurvalanol A (CDK1/2/5)
II-10	AMPK Inhibitor Compound C
II-11	Aurora Kinase Inhibitor III
II-14	Aurora Kinase/CDK Inhibitor
II-15	Indirubin-3′-monoxime (CDK1/GSK3β)
II-16	BAY 11–7082 (IKK)
II-17	Bohemine (CDK)
II-18	Cdk1 Inhibitor
II-19	Cdk1 Inhibitor, CGP74514A
II-20	Cdk1/2 Inhibitor III
II-21	Cdk1/5 Inhibitor
II-22	Casein Kinase I Inhibitor, D4476
II-23	Casein Kinase II Inhib. III, TBCA
II-26	Cdk4 Inhibitor
II-27	Cdk4 Inhibitor II, NSC 625987
II-28	Cdk4 Inhibitor III
II-29	Cdc2-Like Kinase Inhibitor, TG003
II-30	Chk2 Inhibitor II
II-31	Compound 52 (Cdc28p)
II-32	Cdk2 Inhibitor III
II-33	Cdk2 Inhibitor IV, NU6140
II-34	Cdk/Crk Inhibitor
II-35	ERK Inhibitor III
II-38	ROCK Inhibitor, Y-27632
II-39	ERK Inhibitor II, FR180204
II-40	ERK Inhibitor II, Neg. control
II-41	Fascaplysin, Synthetic (CDK4/D1)
II-42	GSK-3b Inhibitor I
II-43	GSK-3b Inhibitor II
II-44	GSK-3b Inhibitor VIII
II-45	GSK-3 Inhibitor IX
II-46	GSK-3 Inhibitor X
II-47	GSK-3b Inhibitor XI
II-50	SU6656 (Src)
II-51	GSK-3 Inhibitor XIII
II-52	Isogranulatimide (CHK1/GSK3β)
II-53	IC261 (Casein kinase 1δ/1ε)
II-54	IKK-2 Inhibitor IV
II-55	Indirubin Derivative E804 (CDC2,CDK2,Src)
II-56	JNK Inhibitor II
II-57	JNK Inhibitor, Negative Control
II-58	JNK Inhibitor V
II-59	JNK Inhibitor IX
II-62	MK2a Inhibitor
II-63	JNK Inhibitor VIII
II-64	K-252a, Nocardiopsis sp. (multiple kinases)
II-65	Kenpaullone (CDK1/2/5)
II-66	KN-93 (CaKKII)
II-67	MEK Inhibitor I
II-68	MEK Inhibitor II
II-69	MEK1/2 Inhibitor
II-70	MNK1 Inhibitor
II-71	NF-kB Activation Inhibitor
II-74	p38 MAP Kinase Inhibitor III
II-75	p38 MAP Kinase Inhibitor
II-76	PD98059 (MEK1/2)
II-77	PD169316 (p38)
II-78	SB220025 (p38)
II-79	Purvalanol A (CDK1/2/5/7)
II-80	GSK3b Inhibitor XII, TWS119
II-81	H-89, Dihydrochloride (S6K1, MSK1, PKA, Rho kinase II)
II-82	SB202474 (neg. control p38 Inhibitor)
II-83	SB202190 (p38)
II-86	SB203580 (p38,AKT)
II-87	HA1077, Dihydrochloride Fasudil (Rho kinase)
II-88	SB218078 (CHK1)
II-89	SC-68376 (p38)
II-90	SKF-86002 (p38)
II-91	Sphingosine Kinase Inhibitor
II-92	Staurosporine (multiple kinases)
II-93	STO-609 (CaM-KK)
II-94	SU9516 (CDK1/2/9)
II-95	TPL2 Kinase Inhibitor

## Discussion

We developed an *in vitro* screening method to identify compounds with the potential to influenced ME mucosal hyperplasia which is induced by bacterial infection. Out of 160 kinase inhibitors screened in triplicate, 38 compounds demonstrated an inhibitory effect on mucosal explants growth, while 2 compounds enhanced growth. The large number of kinase inhibitors that influenced explant growth testify to the complex regulation of ME mucosal hyperplasia by this important group of cellular mediators [27,28].

### Inhibitors reducing mucosal growth

#### Growth factor receptor inhibitors

Inhibitors of both epithelial growth factor (EGF) and platelet derived growth factor (PDGF) receptors reduced mucosal growth. The fact that inhibitors of growth factor receptors were identified in the screen was expected and consistent with prior data. This is particularly the case for EGFR inhibitors, given that the mucosa is an epithelial structure [29], and we have previously shown that heparin-binding EGF (HB-EGF) strongly stimulates the growth of ME epithelial cells in culture [30]. Moreover, the expression of HB-EGF in the ME increases by 26-fold and EGFR expression by 2.5-fold, during OM [25]. Similarly, stromal cells within the explants can be expected to express PDGF receptors, although it has also been found that epithelial cells can respond to injury by up-regulating PDGFRs [31]. During OM, ME PDGFA expression rises 7-fold, and PDGFR expression increases 6-fold [25]. The FBS present in the culture media would supply both EGFs and PDGFs, and production by cells of the explant is also possible. It should also be noted that both EGFRs and PDGFRs can be activated independent of ligands, by reactive oxygen species [32]. Since reactive oxygen species is often released from tissue in culture [33], this provides another potential mode of receptor activation.

#### PI3K/AKT/MTOR inhibitors

Inhibitors of protein kinase B (AKT), phosphatydlinositol 3 kinase (PI3K) and mammalian target of rapamycin (MTOR) reduced explant growth, which was also an expected result. PI3K activation of AKT occurs downstream from the action of many growth factor receptors, and has long been associated with cell proliferation [28]. Substrates of PI3K/AKT signaling are known to be involved in OM. For example, the Class I PI3K catalytic MTOR subunit genes *pik3cd* and *pik3cg* are upregulated 8.5-fold and 2.5-fold, respectively, while the expression of *akt1* increases 3.5-fold, and that of *akt2* and *akt3* increases 2-fold, during OM [25]. A different AKT inhibitor was found to reduce the growth of ME mucosal explants by Lee et al. [34]. Interestingly, these authors also found that an inhibitor of MTOR, which typically opposes PI3K, also reduced mucosal growth. This suggests an atypical role for MTOR in the ME mucosa.

#### Cell cycle progression inhibitors

Of course, it is obvious that cell division is involved in ME mucosal proliferation. It was, therefore, no surprise that several cyclin-dependent kinase (CDK) inhibitors of the cell cycle reduced the growth of ME explants. Similarly, aurora kinases are essential for cell division *via* the regulation of mitosis, especially the process of chromosomal segregation. Thus, suppression of mucosal growth by an aurora kinase A inhibitor was not unexpected.

#### JNK inhibitor

The mitogen-activated protein kinases (MAPKs) are often associated with tissue growth, as in cancer. Activation of Jun kinase (JNK), a cell stress protein, can have many effects, from cell proliferation to apoptosis [35]. During OM, while genes encoding JNK isoforms are only modestly (<2-fold) or not upregulated, expression of the genes encoding the JNK targets cJun and JunB increase 9-fold and 12.6-fold, respectively [25]. JNK inhibition by SP00165 has previously been shown to reduce mucosal growth both *in vitro* and *in vivo* [20], which is why it was chosen as a positive control for this screen. A study in transgenic mice found that lack of the *jnk1* or *jnk2* gene altered the course of bacterial OM [36].

#### NFκB inhibitors

Nuclear factor kappa-B (NF-κB) is a dimeric transcription factor that regulates immune and inflammatory genes in response to infection [37,38]. It also promotes cell proliferation and survival, in part by altering cell cycle regulation (e.g. [39,40]). Activation of NF-κB is tightly regulated, and inappropriate activation of the NF-κB has been linked to autoimmunity, chronic inflammation and cancer [41].

The canonical NF-κB activation pathway, which can be initiated by activated growth factor receptors via PI3K and AKT, relies upon the phosphorylation of IκBα, mediated by a heterotrimer of IκB kinases (IKKs), IKKα, IKKβ and IKKγ [42]. This initiates IκB cleavage of NF-κB1 of a RelA/ NF-κB1 dimer, releasing RelA/p50 to activate expression of genes driving inflammation and tissue growth. A non-canonical NF-κB pathway is activated primarily by members of the TNF receptor family. It activates NF-κB1 independently of the IKK complex to induce persistent activation of RelB/p52. Genes encoding NF-κB and its signaling molecules are strongly upregulated during OM, and inhibition of either the canonical or non-canonical pathways reduces ME mucosal growth *in vitro* [43]. Thus reduction of growth by NF-κB inhibition was expected.

#### PKR inhibitor

An inhibitor of protein kinase R (PKR) reduced explant growth. PKR, a multifunctional kinase best known for being activated by viral double-stranded RNA, can also be activated by the eukaryotic protein PRKRA, and by heparin [44]. Activation of PKR suppresses protein synthesis, and it is known to be involved in the regulation of tissue proliferation via complex interactions with NFκB and the JNK and p38 MAPKs [45]. The gene encoding PKR is modestly (1.5-2X) upregulated during OM, but the gene of its activator PRKRA is strongly suppressed (to 0.4X) within hours after ME infection [25]. PKR has not previously been linked to OM.

#### CAMKII inhibitor

An inhibitor of Ca^++^/calmodulin-dependent protein kinase II (CAMKII) reduced mucosal growth. CAMKIIA is best known for regulating memory and long-term potentiation in the brain [46]. However, CamKII members, especially CAMKIID, have been found to be upregulated in cancers [47], potentially linking them to tissue growth. During OM, while the *Camk2a* gene is strongly (0.15X) *down-regulated*, *Camk2d* is up-regulated 3.4X [25]. CamkIIs have not previously been linked to OM.

#### JAK3 inhibitor

JAK3 is best known for its involvement in signaling via type 1 cytokine receptors, and for expression in leukocytes [48]. However, JAK3 is expressed in a variety of nonlymphoid tissues [49], and several cytokines can be produced by epithelial cells [50]. While it has not been linked previously to ME mucosal hyperplasia, JAK3 has been shown to regulate lung epithelial cell proliferation [51].

### Inhibitors increasing mucosal growth

#### MEK inhibitor II

Library II inhibitor 68, MEK Inhibitor II, significantly enhanced the growth of ME mucosa explants at 0.5 μM. MAPK kinase (MEK) is best known for downstream activation of the ERK MAPK, which is a potent mediator of tissue growth and cell survival [52]. While the *erk1* and *erk1* genes are upregulated only 2-fold during OM, the gene encoding the ERK upstream activator, TPL2, is upregulated more than 35-fold [25]. It has previously been shown that MEK/ERK inhibition by a different inhibitor, U0126, reduces the growth of ME mucosal explants [17]. Therefore, the fact that MEK Inhibitor II increased ME mucosal growth was unexpected. However, while U1026 is selective for MEK1 AND MEK2, it has been shown that MEK Inhibitor II also inhibits TPL2 [53], an upstream activator of several MAPKs and a tumor growth suppressor gene, at a lower IC50 than its inhibition of MEK, which is limited to MEK1 at an IC50 well above 0.5 μM. Inhibition of the growth suppression activity of TPL2 would be consistent with the effects of MEK Inhibitor II on explant growth. Two other MEK inhibitors had no effect on explant growth.

#### H-89 hydrochloride

**Fig 6:**
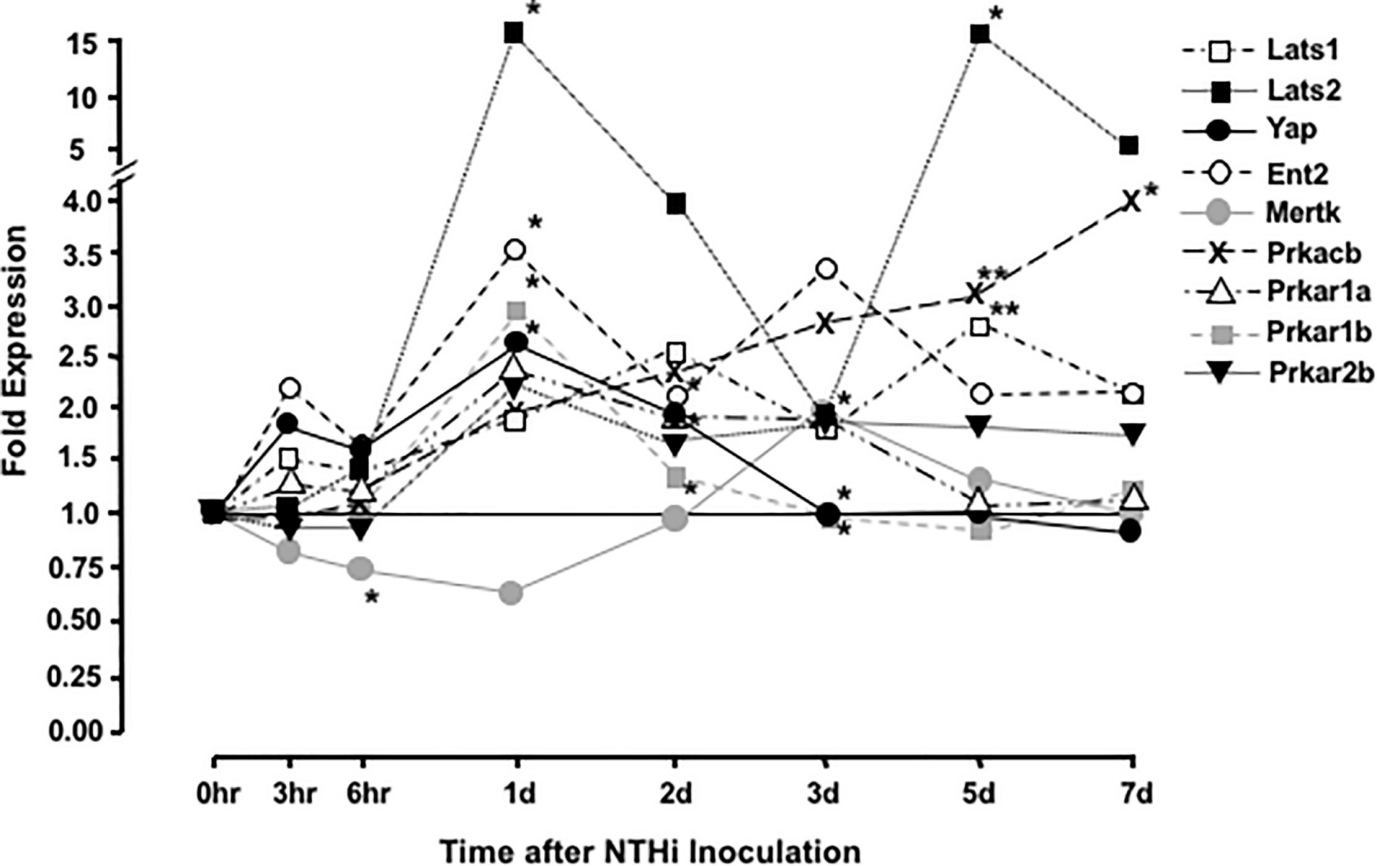
Regulation of Hippo pathway genes in the ME during a complete episode of NTHi-induced acute OM. Gene array analysis of the ME transcriptome reveals that many genes related to Hippo signaling are significantly upregulated beginning 1 day after bacterial inoculation of the ME. * = p< .05; ** = P < .01. The fold change values, ranges and significance levels are represented in S1 Table.

Library II inhibitor 81, H-89 hydrochloride, a potent inhibitor of protein kinase A (PKA), also enhanced explant growth. PKA has been linked to the Hippo signaling pathway, which regulates organ size. Hippo signaling stimulates cell proliferation at low cell densities by directly activating the transcription factor YAP. However, in conditions of cell crowding, the Hippo receptor FAT4 stimulates intermediary proteins including ENT1/2, MERTK, and MST1/2 to activate LATS1/2, which phosphorylates YAP and prevents it from entering the nucleus [54]. PKA is an alternative activator of LATS1/2 [55], and thus can downregulate cell proliferation even in the absence of cell crowding. As shown on Fig 6, a number of Hippo pathway genes are regulated during OM, suggesting the involvement of this pathway in limiting mucosal growth. The Hippo pathway has not previously been identified as having a role in ME mucosal hyperplasia.

### Implications of kinase screen results for mechanisms of ME mucosal hyperplasia

Comparing the results of this screen with Fig 1 and Table 1, it is clear that kinases which participate in the regulation of ME mucosal growth span the breadth of mammalian kinases. Evaluating the targets of inhibitors with the strongest effects on mucosal growth *in vitro*, our results identify three primary kinome groups; the AGC, CMGC, and TK families. The AGC group contains many core intracellular signaling kinases such as PKA, PKG and PKC, after which the family is named, and AKT. AGC kinases are modulated by phospholipids, cyclic nucleotides, and calcium. In various cell types they are responsible for cellular metabolism, transcription regulation, immune responses and cell growth. The CMGC group (also named after the initials of some members) regulates primarily the cell cycle, MAPK signaling, and RNA splicing. Inhibitors identified to have a significant regulatory effect on ME mucosa tended to target Cdks, as well as the JNK and p38 MAPKs from this family. The TK family (the members of which phosphorylate primarily the tyrosine residues of target proteins) are perhaps the most studied kinase group. They have been implicated in a wide variety of cellular processes including cell cycle control, mitogenesis, tissue growth, and cell proliferation. Many growth factor receptors and oncogenes are the primary targets of TK family inhibitors in the ME mucosa.

**Fig 7:**
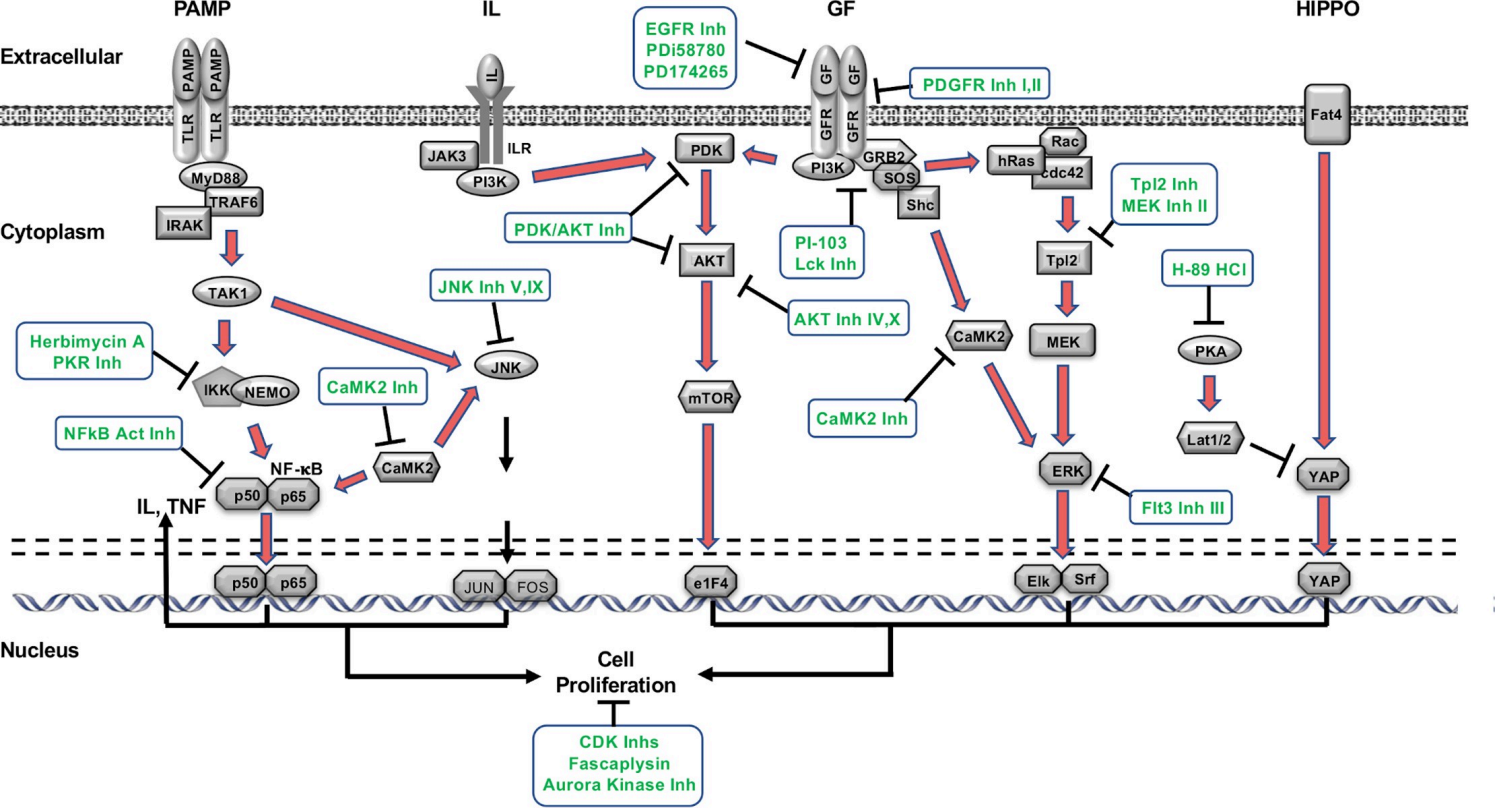
The regulation of ME mucosal growth by kinase pathways. A schematic diagram of signaling pathways identified by the screen as influencing the growth of the ME mucosa in culture. The relevant inhibitors for each pathway are indicated.

This novel *in vitro* screening method identified a number of protein kinases and intracellular signaling pathways which are already known to regulate ME mucosal hyperplasia, as well as additional kinases and pathways not previously associated with mucosal growth. A schematic summary of the kinase pathways regulating tissue growth that were implicated in this screen is provided in Fig 7. This global model provides a rich source of molecules to be evaluated *in vivo*, to confirm any role in the disease process of OM and to evaluate potential therapeutics that could reduce OM pathogenesis.

Explants from infected MEs were used for this screen. We previously noted that explants harvested 48 hours after infection of the ME with NTHi grow significantly faster than explants from naïve MEs. Unlike *in vivo* OM, when the mucosa returns to quiescence after bacteria are cleared, this accelerated *in vitro* growth continues for the duration of culture up to 10 days [18,19]. The reason for the extended growth of previously infected mucosal explants is not clear. The reduction in explant growth by a JAK3 inhibitor suggests that interleukin expression continues in culture for previously infected explants, which may contribute to continuous growth. Similarly, the effects of GF receptor inhibitors indicate that these receptors are also constitutively expressed at high levels in culture and could contribute to prolonged growth. Finally, processes that operate during OM recovery *in vivo* presumably act to suppress interleukin, GF, GF receptor and other growth stimulant expression in mucosal cells. In culture, these suppressive mechanisms may be less active, leading to prolonged growth.

### Advantages and disadvantages of the screening assay

In previous studies, we used ME mucosal explants to evaluate specific growth pathways suspected of mediating mucosal hyperplasia. These and other studies have identified several mechanisms with the potential to influence mucosal growth, including growth factors and downstream signaling pathways. The results of the present study validate the use of ME mucosa explants as a screening tool, with which to broadly evaluate the effects of pharmacological compounds on mucosal growth.

Screening assays have the advantage of being able to test the effects of much larger numbers of compounds than more detailed i*n vivo* studies. This allows for the evaluation of compounds without *a priori* assumptions regarding the mechanism underlying mucosal growth, and permits the discovery of novel cellular mechanisms not predicted from current knowledge. In the case of this screen, several such previously unsuspected mechanisms were uncovered, and several novel inhibitors were identified. A throughput screening assay also provides comparative information on the relative ability of compounds to inhibit growth. Thus, we found that some compounds with similar kinase targets displayed quite disparate inhibitory ability.

However, the nature of screening means that the assay used is constrained by necessity. For most screens, as in our case, *in vitro* methods are necessary to increase throughput. Dissection and tissue culture can alter the responses of cells and tissue. In addition, rodents are separated by millions of years of evolution from humans, and may exhibit significant differences. That said, murine and human MEs exhibit more similarities than differences. There have been many studies demonstrating that the ME mucosa from both species respond similarly to bacterial infection (e.g. [16,56]), while genetic defects that influence OM in the mouse often have similar effects on human disease (e.g. [57]).

Another issue for the assay is that only a limited number of conditions can be evaluated. ME mucosal explants cannot be produced in very large numbers, as is possible with cell lines. While we used three different concentrations of the screened compounds, dosages outside of this range might also have proved effective. This study was performed as a screen, and testing a larger number of dosages would have made the screen less practical. The effects of compounds that might require a longer pre-treatment period may have also been missed.

As noted above, several of the pathways identified in our screen have previously been evaluated using inhibitors or gene deletion models. These prior studies provide retrospective validation of these assay hits. However, the novel pathways identified require confirmation by more extensive studies and evaluation *in vivo*.

### Potential clinical implications

The results of the present study have implications for the treatment of OM in patients. As noted above, OM is characterized by extensive hyperplasia of the ME mucosa. The transformation of the mucosa from a monolayer of simple squamous epithelium to a pseudostratified, cuboidal epithelium with multiple cell layers and the characteristics of a respiratory epithelium [16] can result in many deleterious sequelae. This includes the generation of mucus from goblet cells, the secretion of additional factors from secretory cells, and the growth of a supportive vasculature. Mucus can result in reduced ability to clear fluid from the ME cavity. In chronic OM, this can result in “glue ear” with mucus and cellular debris so dense that clearance is virtually impossible [58]. Neovascularization can enhance the entry of inflammatory leukocytes into the ME, as well as the extravasation of fluid into the tympanic cavity, resulting in cellular effusion.

We have identified pathways that are likely responsible for hyperplasia of the ME mucosa, as well as inhibitors that are capable of limiting its growth. These and similarly targeted inhibitors have the potential to reduce the above damaging sequelae of OM, and reduce pathogenesis. Most uncomplicated OM resolves without treatment over the course of days [8], and would not be targets for intervention. However, chronic OM could benefit from limiting mucosal growth. Obviously, hyperplasia of the mucosa, mucus generation and leukocyte infiltration can serve a protective function against bacterial infection of the ME. However, antibiotics are prescribed for chronic OM [11], and the response of the ME mucosa can be deleterious. In such cases, mucosal growth inhibitors may have a place alongside antimicrobial therapy.

## Supporting information

S1 Table Fold change averages, ranges and probabilities for gene array data presented in Fig 6. (DOC)

S1 Fig (JPG)
