# Supplementary material for: A kinase inhibitor screen identifies signaling pathways regulating mucosal growth during otitis media
Source: PLoS One. 2020 Aug 6;15(8):e0235634. doi: 10.1371/journal.pone.0235634 (PMC7410257; doi:10.1371/journal.pone.0235634)
Supplement: S1 Table — (DOC) [file pone.0235634.s001.doc]

**PKA genes**

PRKACB 1420610_at Prkacb

Time 0.0 0.805 (0.406 to 1.594) 0.804

Time 3.0 0.994 (0.876 to 1.126) 0.967

Time 6.0 1.133 (0.725 to 1.771) 0.826

Time 24.0 1.835 (1.643 to 2.049) 0.114

Time 48.0 2.117 (1.604 to 2.795) 0.226

Time 72.0 2.606 (2.124 to 3.197) 0.134

**Time 120.0 2.976 (2.966 to 2.987) 0.00208**

**Time 168.0 3.719 (3.483 to 3.972) 0.0318**

PRKAR1A 1425550_a_at Prkar1a

Time 0.0 1 (0.999 to 1.001) 1

Time 3.0 1.304 (1.28 to 1.329) 0.0456

Time 6.0 1.194 (1.11 to 1.285) 0.249

**Time 24.0 2.285 (2.196 to 2.377) 0.0304**

Time 48.0 1.773 (1.568 to 2.005) 0.135

Time 72.0 1.775 (1.614 to 1.952) 0.105

Time 120.0 1.12 (1.108 to 1.132) 0.0597

**Time 168.0 1.178 (1.166 to 1.19) 0.0392**

PRKAR1B 1416754_at Prkar1b

Time 0.0 0.735 (0.322 to 1.678) 0.773

Time 3.0 1.06 (0.901 to 1.248) 0.78

Time 6.0 1.147 (0.959 to 1.372) 0.584

**Time 24.0 2.927 (2.768 to 3.095) 0.033**

Time 48.0 1.348 (1.28 to 1.419) 0.109

Time 72.0 0.972 (0.682 to 1.386) 0.949

Time 120.0 0.895 (0.695 to 1.153) 0.737

Time 168.0 1.236 (0.855 to 1.789) 0.668

PRKAR2B 1438664_at Prkar2b

Time 0.0 0.989 (0.855 to 1.145) 0.954

Time 3.0 0.715 (0.693 to 0.738) 0.0602

Time 6.0 0.772 (0.555 to 1.075) 0.578

**Time 24.0 0.292 (0.268 to 0.318) 0.0439**

Time 48.0 0.616 (0.563 to 0.674) 0.117

Time 72.0 0.545 (0.371 to 0.8) 0.359

Time 120.0 1.138 (0.972 to 1.332) 0.564

Time 168.0 0.717 (0.654 to 0.785) 0.17

**Hippo Pathway Genes**

Slc29a2 1448257_at Slc29a2

Time 0.0 1 (0.991 to 1.009) 0.997

Time 3.0 2.177 (1.94 to 2.443) 0.0937

Time 6.0 1.725 (1.058 to 2.812) 0.465

Time 24.0 3.425 (2.523 to 4.651) 0.155

**Time 48.0 1.906 (1.818 to 1.999) 0.0466**

**Time 72.0 3.159 (2.93 to 3.406) 0.0416**

Time 120.0 1.841 (1.735 to 1.953) 0.0617

Time 168.0 1.912 (1.3 to 2.81) 0.341

Mertk 1422869_at Mertk

Time 0.0 0.976 (0.784 to 1.216) 0.931

Time 3.0 0.816 (0.563 to 1.185) 0.682

**Time 6.0 0.695 (0.681 to 0.71) 0.0365**

Time 24.0 0.571 (0.502 to 0.65) 0.144

Time 48.0 0.964 (0.763 to 1.219) 0.902

**Time 72.0 1.931 (1.93 to 1.933) 0.000697**

**Time 120.0 1.19 (1.179 to 1.2) 0.0321**

Time 168.0 1 (0.854 to 1.17) 0.998

Mst2 1418513_at Stk3

Time 0.0 0.989 (0.854 to 1.146) 0.953

Time 3.0 0.903 (0.785 to 1.039) 0.599

Time 6.0 0.907 (0.756 to 1.088) 0.687

Time 24.0 2.032 (1.881 to 2.195) 0.0691

Time 48.0 1.512 (1.463 to 1.563) 0.0504

Time 72.0 1.762 (1.685 to 1.843) 0.0502

Time 120.0 1.657 (1.485 to 1.848) 0.136

Time 168.0 1.55 (1.346 to 1.783) 0.198

Lats1 1427679_at Lats1 ---

Time 0.0 1 (0.972 to 1.028) 0.991

Time 3.0 1.496 (1.223 to 1.83) 0.295

Time 6.0 1.36 (0.935 to 1.98) 0.563

Time 24.0 1.739 (1.183 to 2.558) 0.388

Time 48.0 2.459 (2.077 to 2.912) 0.118

Time 72.0 1.677 (1.596 to 1.763) 0.0612

**Time 120.0 2.63 (2.606 to 2.654) 0.00599**

Time 168.0 2.077 (1.522 to 2.835) 0.256

Lats2 1419679_at Lats2 ---

Time 0.0 0.988 (0.845 to 1.155) 0.951

Time 3.0 1.092 (0.974 to 1.225) 0.582

Time 6.0 1.291 (0.895 to 1.864) .613

**Time 24.0 15.55 (14.25 to 16.96) 0.0202**

Time 48.0 4.431 (2.787 to 7.043) 0.192

Time 72.0 1.851 (1.241 to 2.76) 0.366

**Time 120.0 15.33 (14.49 to 16.21) 0.0131**

Time 168.0 6.55 (3.458 to 12.4) 0.209

Yap 1416487_a_at Yap ---

Time 0.0 0.996 (0.906 to 1.094) 0.97

Time 3.0 1.785 (1.461 to 2.182) 0.212

**Time 6.0 1.576 (1.546 to 1.607) 0.0272**

**Time 24.0 2.589 (2.444 to 2.744) 0.0387**

Time 48.0 1.71 (1.541 to 1.898) 0.122

Time 72.0 0.96 (0.89 to 1.035) 0.683

Time 120.0 0.979 (0.907 to 1.057) 0.825

Time 168.0 0.863 (0.843 to 0.884) 0.101

Tead4 1426337_a_at Tead4 ---

Time 0.0 0.966 (0.742 to 1.258) 0.917

Time 3.0 0.851 (0.424 to 1.707) 0.855

**Time 6.0 2.191 (2.149 to 2.234) 0.0158**

Time 24.0 3.488 (3.143 to 3.872) 0.053

Time 48.0 0.807 (0.379 to 1.72) 0.824

Time 72.0 0.598 (0.361 to 0.991) 0.494

Time 120.0 0.786 (0.414 to 1.491) 0.771

Time 168.0 0.379 (0.346 to 0.415) 0.0596

Fat4 – N.R.
