# Supplementary figures and images for: A kinase inhibitor screen identifies signaling pathways regulating mucosal growth during otitis media
Source: PLoS One. 2020 Aug 6;15(8):e0235634. doi: 10.1371/journal.pone.0235634 (PMC7410257; doi:10.1371/journal.pone.0235634)

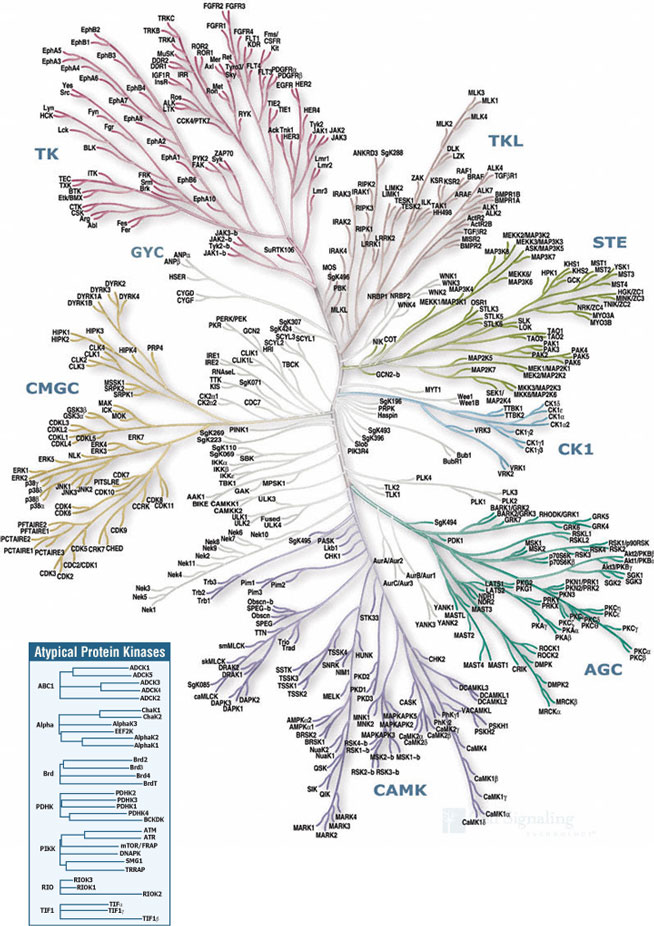

Supplement: S1 Fig — (JPG) [file pone.0235634.s002.jpg]
